# The Effects of Acupuncture Treatment on Sleep Quality and on Emotional Measures among Individuals Living with Schizophrenia: A Pilot Study

**DOI:** 10.1155/2013/327820

**Published:** 2013-09-03

**Authors:** Alon Reshef, Boaz Bloch, Limor Vadas, Shai Ravid, Ilana Kremer, Iris Haimov

**Affiliations:** ^1^Psychiatric Department, Emek Medical Center, Afula, Israel; ^2^Technion—Israel Institute of Technology, Haifa, Israel; ^3^Department of Psychology and the Center for Psychobiological Research, Yezreel Academic College, Emek Yezreel 19300, Israel; ^4^Mazra Mental Health Center, Akko, Israel

## Abstract

*Purpose*. To examine the effects of acupuncture on sleep quality and on emotional measures among patients with schizophrenia. *Methods*. Twenty patients with schizophrenia participated in the study. The study comprised a seven-day running-in no-treatment period, followed by an eight-week experimental period. During the experimental period, participants were treated with acupuncture twice a week. During the first week (no-treatment period) and the last week of the experimental period, participants filled out a broad spectrum of questionnaires and their sleep was continuously monitored by wrist actigraph. *Results*. A paired-sample *t*-test was conducted comparing objective and subjective sleep parameters manifested by participants before and after sequential acupuncture treatment. A significant effect of acupuncture treatment was observed for seven objective sleep variables: sleep onset latency, sleep percentage, mean activity level, wake time after sleep onset, mean number of wake episodes, mean wake episode and longest wake episode. However, no significant effects of acupuncture treatment were found for subjective sleep measures. Likewise, the results indicate that acupuncture treatment improved psychopathology levels and emotional measures, that is, depression level and anxiety level. *Conclusions*. Overall, the findings of this pilot study suggest that acupuncture has beneficial effects as a treatment for insomnia and psychopathology symptoms among patients with schizophrenia.

## 1. Introduction

Schizophrenia is a mental disorder involving disturbances in basic mental functions, such as emotions, cognition, perception, and other aspects of behavior [1–3]. The clinical picture of schizophrenia is characterized by a mixture of two main categories of core symptoms: positive symptoms (i.e., delusions and hallucinations) and negative symptoms (i.e., apathy, flat affect, and lack of functioning) [4–6]. Its lifetime prevalence is about 1%, with equal distribution between men and women [4]. Schizophrenia is known to be one of the most debilitating and distressful mental disorders, and during its course the disease often deteriorates and becomes chronic [7–9]. Schizophrenia is responsible for tremendous emotional and economic burdens on patients, their families, and society as a whole [8, 10].

Further, it may be valuable to consider schizophrenia as a multidimensional disorder that includes several different domains, among them clinical, neurocognitive, occupational, familial, and societal, and perhaps most important subjective well-being and positive feelings [11]. Besides the core symptoms mentioned above, other clinical expressions of schizophrenia include depressive features, anxiety features and sleep disturbances, of which insomnia is most prevalent [12–14]. The treatment of schizophrenia is complex and should be tailored to the patient, taking into consideration biopsychosocial components. Yet standard treatments, especially biological treatments, are only partially effective and have possible serious side effects. 

Insomnia is the most common sleep disorder in psychiatry [15, 16]. Polysomnographic studies have shown that patients with schizophrenia exhibit increased sleep latency manifested by difficulties initiating or maintaining sleep, reduced sleep efficiency and total sleep time, reduced REM latency and REM density, and disturbed non-REM sleep architecture, specifically a decrease in SWS, especially in stage 4 [12, 17–21]. Among patients with schizophrenia, insomnia may precede relapse or appear during exacerbated schizophrenic episodes; it may even complicate schizophrenia to the degree that patients can exhibit suicidal behavior [22, 23]. 

Many studies have provided evidence showing that insomnia is not simply a typical symptom of depression or of other psychiatric disorders but may actually be a predictor (or an independent risk factor) for the development of such conditions [24, 25]. Further research has indicated that insomnia can be an important indicator in the diagnosis of mental disorder and can also play a role in the deterioration of existing mental disorders [25, 26]. Moreover, Kantrowitz et al. proposed in their study that sleep deficits reflect a core element of schizophrenia [20]. 

Among patients with schizophrenia, sleep disturbances and, specifically, changes in sleep architecture and sleep quality have an enormous impact on quality of life, level of functioning, and cognitive functioning [20]. Hence, preserving the integrity of sleep among such patients is an important health priority. The most prevalent treatment for insomnia in patients with schizophrenia involves pharmacotherapy. Medication has many disadvantages, among them side effects that add emotional, social, and economic burdens to the existing burden of the disease. For instance, one of the most common treatments for insomnia is the use of benzodiazepines, a heterogeneous class of anxiolytic drugs distinguished by their half-lives. At high doses, benzodiazepines result in across-the-board side effects, notably memory impairment, drowsiness, and increased likelihood of accidents [27]. Another common drug treatment is the use of imidazopyridines, whose effects have been found to resemble those of short-acting benzodiazepines [28–30] but with less influence on sleep architecture, cognitive behaviors, and withdrawal symptoms. In addition, antidepressants, antihistamines, and antipsychotics [31] have been mentioned as treatments for insomnia. Many of these have also been determined to have serious side effects, particularly during prolonged use [32]. Additionally, all drug treatments are relatively contraindicated in pregnancy, sleep apnea, liver or kidney dysfunction, prior history of substance abuse, or certain jobs requiring atypical shifts [27–32].

The disadvantages of drug treatment for insomnia in patients with schizophrenia underline the importance of seeking alternative nonpharmacological treatments, among them complementary medicine techniques such as shiatsu, reflexology, and acupuncture. Moreover, despite recent achievements in psychopharmacology, the pharmacological treatment for schizophrenia is far from satisfactory. Hence, additional treatment modalities are most welcome. Complementary therapies are widely used among psychiatric patients, including patients with schizophrenia. In the western world and in Israel, the most popular modality used for many somatic and mental disorders is acupuncture. Cumulative data suggest that complementary treatment strategies, including acupuncture, can be applied and carefully examined in schizophrenia [33–36].

Acupuncture is one of the oldest healing practices in the world. It is considered to be a safe and effective treatment modality which, according to Traditional Chinese Medicine (TCM), harmonizes the body's energies [37]. Acupuncture incorporates the use of ultrafine needles (0.15–0.30 mm diameter) inserted into specific points on the skin (acupoints). TCM teaches that the body's energy, or qi (pronounced chee), flows along a series of points called meridians. Each internal organ has a corresponding meridian, and the application of pressure (acupressure, shiatsu), heat (moxibustion), or needles (acupuncture) to the relevant acupoints is believed to have an impact upon the associated internal organs and thus to harmonize the body's qi [38]. The exact mechanism by which acupuncture induces physiological changes, relieves pain, and alleviates illness is still unclear. Research has shown that treatment with acupuncture results in nonspecific (placebo response) as well as specific effects such as local and systemic effects, among them an increase in the release of pituitary beta-endorphins and ACTH [38]. This release of endorphins may partly explain the analgesic effects of this treatment, whereas increased ACTH secretion—which leads to elevated serum cortisol levels—may account for its anti-inflammatory effects. Acupuncture can also cause accelerated synthesis and release of serotonin and noradrenaline into the central nervous system, thus activating descending antinociceptive pathways and deactivating multiple limbic areas that promote pain association [38].

Previous studies have demonstrated that acupuncture has a positive influence on a number of diseases and disorders, among them depression, chronic pain, and sleep disorders [39–41]. Nevertheless, tests of the use and effectiveness of acupuncture for mental disorders and sleep disturbances have yielded heterogeneous and inconclusive results [40, 42]. One explanation for these results may be the paucity of studies conducted so far, while another may be the lack of objective outcome measures. Objective sleep assessment may therefore be a preferable outcome measure, in addition to the usual clinical assessment tools. Another explanation may be the difficulty in testing Eastern treatments techniques using impeccable Western methodology. 

The correlation between sleep difficulties and schizophrenia along with the data showing the beneficial effects of acupuncture for insomnia suggests that acupuncture may have a positive effect for patients with schizophrenia. Moreover, objective sleep measurement can be an excellent outcome measure and may help answer some validity issues concerning the effectiveness of TCM therapies in schizophrenia. 

Accordingly, the current pilot study examines the effects of the acupuncture technique as a treatment for insomnia and as a possible treatment of core symptoms and emotional measures among patients with schizophrenia.

## 2. Method

The clinical experiment conformed to the principles outlined by the Declaration of Helsinki, and the complete study protocol was approved by the Helsinki Committee of the Haemek Medical Center (number 0015-08-EMC) and by the Institutional Ethics Committee of the Yezreel Academic College of Emek Yezreel (number 0028-YVC). After the study was completely described to all participants, their written informed consent was obtained. 

### 2.1. Participants

Twenty individuals living with schizophrenia or schizoaffective disorder participated in the study (mean age = 43.15, SD = 9.42; 10 males and 10 females). All the patients were diagnosed as being on the schizophrenia spectrum (i.e., schizophrenia and schizoaffective disorder) (ICD-10 criteria) [43] and were treated with antipsychotic medications. In addition, some of them were treated with combinations of antidepressants and mood stabilizers along with anxiolytic medications, a common practice for the treatment of this clinical population [43]. 

Inclusion criteria included ICD-10 diagnosis of schizophrenia spectrum, clinical stability with no medication change during one month prior to study beginning.

Participants were ineligible for inclusion in the current study if they demonstrated symptomatic aggravation, suicidality (suicidal ideation or acts) during one month prior to study beginning, substance abuse, or physical or neurological unstable conditions [43]. 

All participants were recruited from the outpatient clinics of the Department of Psychiatry at Haemek Medical Center. Patients were recruited by a convenience sample since this was a naturalistic pilot design. Patients were consecutive and were not picked at certain days. All participants were living independently in the community or in rehabilitation settings (hostels). All of them had been stable in following their medication regimens for at least one month before the study began. All participants completed the study period; that is, the dropout rate was zero.

### 2.2. Procedure

The study began with a seven-day running-in, treatment-as-usual period, followed by an eight-week experimental period. During the experimental period, participants were treated with acupuncture twice a week, for a total sequential acupuncture treatment comprising 16 sessions. During the first week of the study (treatment-as-usual period) and the last week of the experimental period, participants' sleep was continuously monitored with a wrist actigraph, and participants completed a broad spectrum of questionnaires. 

### 2.3. Measurements

General information was provided by a demographic questionnaire suitable for the study population. Evaluations included psychiatric clinical assessment, emotional condition assessment, sleep assessment, and Chinese medicine assessment. 

#### 2.3.1. Sleep Measurements

*Sleep Questionnaires*. All participants completed three questionnaires that subjectively evaluated their sleep patterns: (i) a qualitative questionnaire—the Mini Sleep Questionnaire (MSQ) [44, 45]; (ii) a quantitative informative questionnaire—the Technion Sleep Questionnaire [46]; (iii) a qualitative questionnaire—the Pittsburgh Sleep Quality Index Hebrew Translation (PSQI-H) [47].

*Wrist Actigraph Recording.* For the purposes of objectively evaluating sleep, each participant's sleep was continuously monitored over a one-week period by a miniature actigraph worn on the wrist (Mini Motionlogger, Ambulatory Monitoring, Inc., Ardsley, NY, USA). The wrist actigraph facilitates monitoring sleep under natural circumstances with minimal distortions. The actigraph measures wrist activity utilizing a piezoelectric element and translates wrist movements into an electrical signal that is digitized and memorized. Activity is recorded at 60-second intervals [48, 49]. The recordings were analyzed by an automatic algorithm (W2 scoring algorithm) provided by the manufacturer to determine time in bed (total number of minutes from bedtime to wake time), sleep onset latency (time taken to fall asleep from bedtime), sleep percentage (percentage of total sleep time out of total time in bed), mean activity level (mean activity score measured in counts/min), wake time after sleep onset (total number of wake minutes after sleep onset), mean number of wake episodes, mean wake episode (mean number of minutes participant is awake during waking episodes after initially falling asleep), and longest wake episode (number of minutes participant is awake during the longest waking episode after initially falling asleep).

#### 2.3.2. Clinical and Emotional Assessment

Participants were interviewed by a psychiatrist and completed two questionnaires aimed at measuring their clinical morbidity.

1. Brief Psychiatric Rating Scale (BPRS) [50, 51].
2. Positive and Negative Syndrome Scale (PANSS) [51, 52] for schizophrenia and schizoaffective patients only.

Participants' level of depression was assessed through two subjective questionnaires.

1. Beck Depression Inventory (BDI) [53, 54] is a 21-item self-report questionnaire. The 21 items correspond to symptoms such as mood, pessimism, and suicidal thoughts. The BDI is an internally consistent and valid measurement [55].
2. Calgary Depression Scale for schizophrenia (CDSS) is a 9-item test that measures the severity of depression. The CDSS is used to assess depressive symptoms in people with schizophrenia, independent of their positive, negative, and general symptoms [56–58]. The questionnaire rates the severity of symptoms observed in depression, such as low mood, insomnia, agitation, anxiety and weight loss.

Anxiety level was assessed by two questionnaires.

1. State-Trait Anxiety Inventory (STAI) [59–61] is a 40-item self-report measure comprising two 20-item scales. The first scale measures state anxiety, defined as a transitory emotional state or condition, and the second measures trait, character, and logical anxiety [60, 61].
2. Hamilton Anxiety Rating Scale (HAS) is a 14-item test that measures the severity of anxiety symptoms in children and adults. It provides measures of overall anxiety, psychic anxiety (mental agitation and psychological distress), and somatic anxiety (physical complaints related to anxiety) [62, 63].

Hedonic state was assessed by the Snaith-Hamilton Pleasure Scale (SHAPS). The SHAPS questionnaire includes 14 items that indicate levels of hedonic states [64, 65]. 

The Quality of Life Enjoyment and Satisfaction Questionnaire (Q-LES-Q) [66, 67] was used to measure participants' general satisfaction with their life.

#### 2.3.3. Chinese Medicine Assessment

All participants were interviewed by an acupuncture therapist at the beginning of each session (i.e., during the entire 16-session sequence) and answered a diagnostic Chinese medicine questionnaire to assess present diagnosis of physical and mental conditions from the perspective of TCM. This questionnaire assesses physical and mental morbidities, and primary and secondary complaints. In addition, at the beginning of each session, participants' pulse and tongue measurements were taken using Chinese medicine techniques.

### 2.4. Acupuncture Treatment

The treatment was acupuncture (needle acupuncture) using standard Chinese needles sized 0.15 mm × 0.18 mm, 0.22 mm × 0.30 mm, and 0.25 mm × 0.40 mm. The use of each size needle and the acupoint corresponding to it were determined by the guidelines set out in the *Manual of Acupuncture* [68]. Each treatment lasted 30 minutes. The acupoints selected by the practitioners were chosen for their compatibility with the practitioners' diagnosis of each patient. The treatment regimen and selected acupoints were determined by traditional Chinese medicine diagnosis of syndromes marked by complex sets of signs and symptoms. These syndromes do not fit Western diagnoses such as depression or schizophrenia. For example, the Western diagnosis of depression can be referred to as “liver qi stagnation” or “blood deficiency” or “heart fire” in traditional Chinese medicine. Certified acupuncturists gave the acupuncture treatments, and each patient had his own acupuncturist for all the sessions of this study. The treatment method and acupoints were chosen according to the patient's diagnosis in an attempt to address each and every patient's individual condition “as a whole” rather than aiming only at the patient's sleeping disorder by using a set of “protocol points.” 

### 2.5. Statistical Analysis

A paired sample *t*-test was conducted comparing psychopathology score, emotional measures, and objective and subjective sleep parameters manifested by participants before and after sequential acupuncture treatment.

## 3. Results

### 3.1. Objective and Subjective Sleep Measures

Actigraphic sleep measures included eight measures of sleep quality: time in bed, sleep onset latency, sleep percentage, mean activity level, wake time after sleep onset, mean number of wake episodes, mean wake episode, and longest wake episode. A paired sample *t*-test was conducted comparing objective sleep parameters manifested by participants before and after sequential acupuncture treatment. A significant effect of acupuncture treatment was observed for seven sleep variables (Table 1).

**Figure 1:**
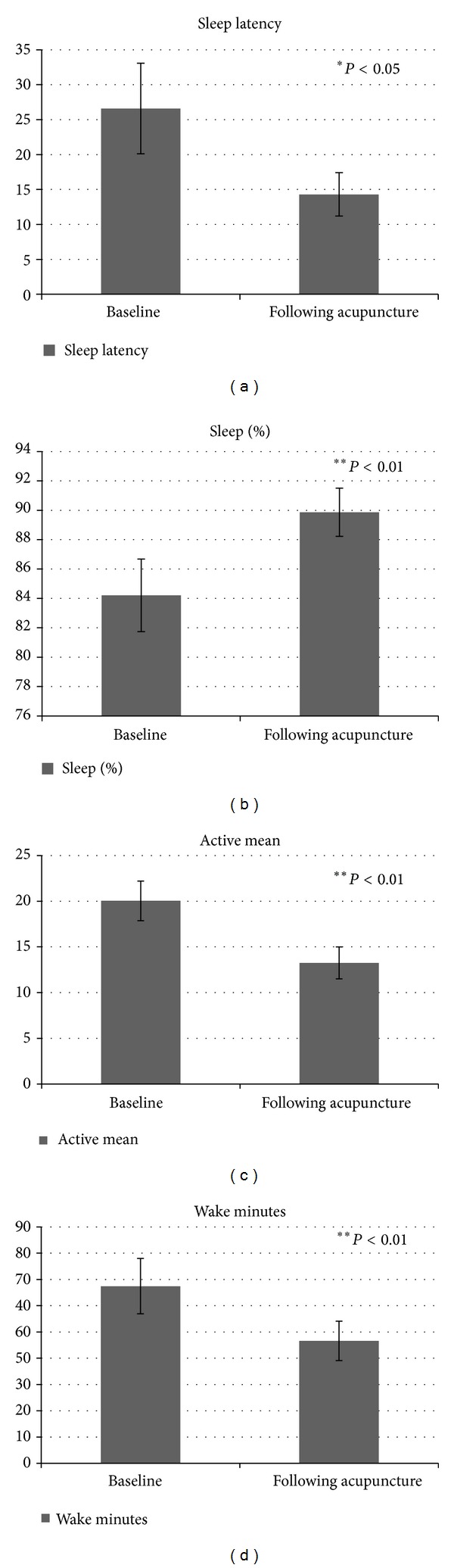
Objective measures of sleep as recorded by actigraph before and after acupuncture treatment: (a) sleep latency, (b) % of sleep percentage, (c) mean activity during sleep, and (d) total wake minutes during sleep.

A significant difference was found in sleep latency (*t*(16) = 2.21, *P* < 0.05), indicating shorter sleep latency following acupuncture treatment (M = 14.32, SD = 12.81; M = 26.63, SD = 26.71, resp.) (Figure 1(a)). In addition, a significant difference was found in sleep percentage (*t*(18) = 2.9, *P* < 0.01), indicating higher sleep percentage following acupuncture treatment (M = 89.86, SD = 7.14) compared with baseline measures (M = 84.20, SD = 10.76) (Figure 1(b)). Likewise, a significant reduction was found in mean activity level (*t*(18) = 3.2, *P* < 0.01). Following acupuncture treatment, participants were less active during sleep compared to baseline measures (M = 13.26, SD = 7.63; M = 20.04, SD = 9.43, resp.) (Figure 1(c)). 

A significant difference was also found in total wake minutes (Figure 1(d)) (*t*(18) = 2.92, *P* < 0.01), indicating fewer total wake minutes during sleep following acupuncture treatment (M = 46.58, SD = 32.64) compared to baseline measures (M = 67.43, SD = 45.86). Similarly, significant differences were found in mean wake episodes, long wake episodes, and longest wake episode (*t*(18) = 2.26, *P* < 0.05; *t*(18) = 2.49, *P* < 0.05; *t*(18) = 2.87, *P* < 0.05, resp.), revealing shorter mean wake episodes (M = 5.42, SD = 2.31), shorter long wake episodes (M = 2.85, SD = 1.54), and shorter longest wake episode (M = 18.16, SD = 11.55) following acupuncture treatment compared to baseline measures (M = 7.21, SD = 4.81; M = 3.76, SD = 1.62; M = 25.71, SD = 19.20, resp.). However, no significant effect of acupuncture treatment was found for total time in bed (n.s.).

A paired sample *t*-test was conducted comparing subjective sleep parameters manifested by participants before and after sequential acupuncture treatment. No significant effects of acupuncture treatment were found for subjective sleep measures (n.s.) (Table 1).

### 3.2. Objective and Subjective Emotional and Clinical Measures

A paired sample *t*-test was conducted comparing psychopathology score manifested by participants before and after sequential acupuncture treatment (Table 2). 

**Figure 2:**
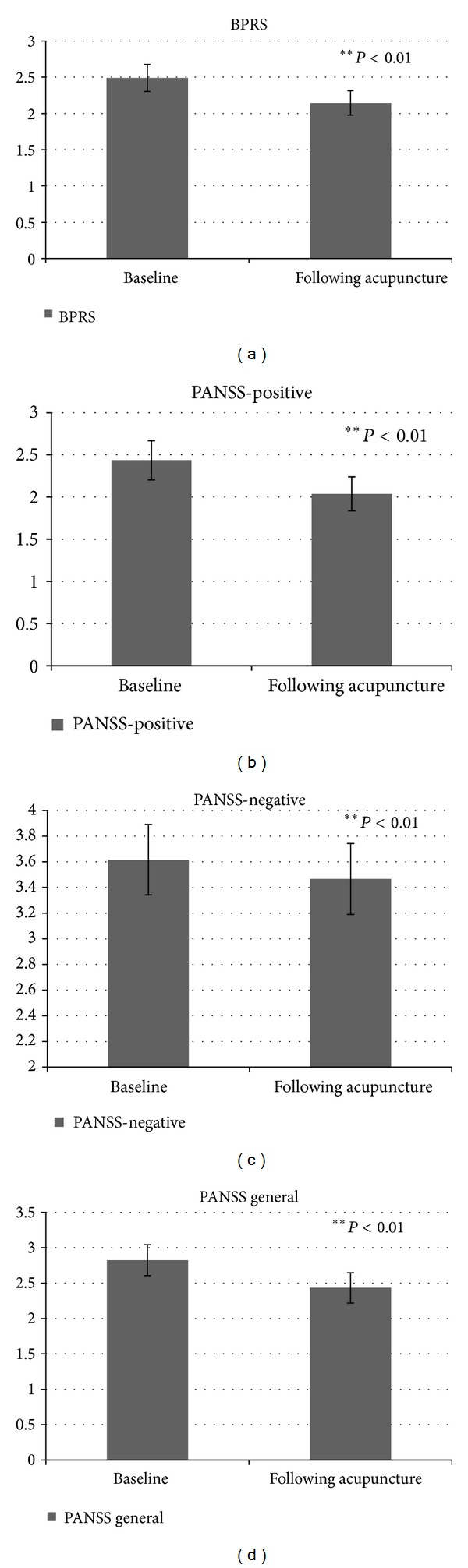
Objective psychopathology levels before and after acupuncture treatment: (a) general psychopathology score as recorded by the Brief Psychiatric Rating Scale (BPRS), (b) positive symptoms as measured by the Positive and Negative Syndrome Scale (PANSS), (c) negative symptoms as measured by the Positive and Negative Syndrome Scale (PANSS), and (d) general symptoms as measured by the Positive and Negative Syndrome Scale (PANSS).

Objective psychopathology levels were measured by the Brief Psychiatric Rating Scale (BPRS) and by the Positive and Negative Syndrome Scale (PANNS). A significant difference was found in psychopathology levels assessed by the Brief Psychiatric Rating Scale (BPRS), indicating that acupuncture improved the general psychopathology score (*t*(18) = 6.67, *P* < 0.01), with a lower general psychopathology score following acupuncture treatment (M = 2.14, SD = 0.74) compared with the baseline score (M =2.49, SD = 0.81) (Figure 2(a)). 

Similarly, the results indicate that acupuncture improved the overall psychopathology score assessed by the Positive and Negative Syndrome Scale (PANNS) (*t*(19) = 8.26, *P* < 0.01), with a lower total psychopathology score following acupuncture treatment (M = 2.65, SD = 0.94) compared with the baseline measure (M = 2.96, SD = 0.98). 

Likewise, significant differences were found for positive, negative, and general symptoms assessed by the Positive and Negative Syndrome Scale (PANNS) (*t*(18) = 5.23, *P* < 0.01; *t*(18) = 2.97, *P* < 0.01; *t*(18) = 7.96, *P* < 0.01, resp.), revealing lower positive, negative, and general symptoms following acupuncture treatment (M = 2.04, SD = 0.88; M = 3.47, SD = 1.20; M = 2.43, SD = 0.93, resp.) compared with baseline measures (M = 2.44, SD = 1.01; M = 3.62, SD = 1.20; M = 2.83, SD = 0.95, resp.) (Figures 2(b), 2(c), and 2(d)). 

A paired sample *t*-test was conducted comparing emotional measures manifested by participants before and after sequential acupuncture treatment (Table 2). 

**Figure 3:**
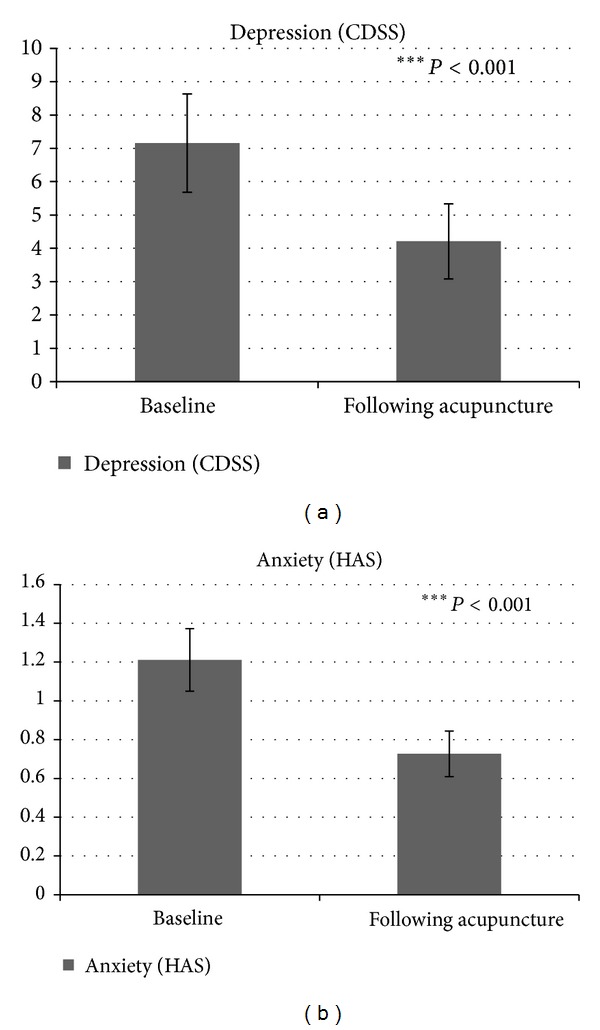
Emotional measures before and after acupuncture treatment: (a) depression level as measured by the Calgary Depression Scale for schizophrenia (CDSS), (b) anxiety level as measured by the Hamilton Anxiety Rating Scale (HAS).

A marginal significant difference was found in depression level as measured by the Beck Depression Inventory (BDI) questionnaire (*t*(19) = 1.79, *P* = 0.088), showing a reduced depression level following acupuncture treatment (M = 12.01, SD = 11.61) compared with the baseline depression level (M = 14.66, SD = 11.89). Moreover, a significant difference was found in depression level as measured by the Calgary Depression Scale for schizophrenia (CDSS) questionnaire (*t*(18) = 5.04, *P* < 0.01), revealing a lower depression level following acupuncture treatment (M = 4.21, SD = 4.90) compared to the baseline depression level (M = 7.16, SD = 6.43) (Figure 3(a)). 

In addition, a significant difference was found in anxiety level as measured by the State-Trait Anxiety Inventory (STAI) and by the Hamilton Anxiety Rating Scale (HAS) questionnaires (*t*(19) = 2.98, *P* < 0.01; *t*(17) = 5.96, *P* < 0.01, resp.), revealing a lower anxiety level following acupuncture treatment (M = 2.12, SD = 0.48; M = 0.73, SD = 0.49) compared with the baseline anxiety level (M = 2.27, SD = 0.52; M = 1.21, SD = 0.68) (Figure 3(b)). 

A paired sample *t*-test was conducted comparing quality of life levels manifested by participants before and after sequential acupuncture treatment. The results revealed a marginal significant difference in quality of life level measured by the Quality of Life Enjoyment and Satisfaction Questionnaire (Q-LES-Q) (*t*(19) = −2.08, *P* = 0.05), showing a higher level of quality of life following acupuncture treatment (M = 3.70, SD = 0.55) compared with the baseline quality of life level (M = 3.60, SD = 0.58). 

However, no significant effects of acupuncture treatment were found for depression level measured by the Beck Depression Inventory (BDI) or for hedonic level measured by the Snaith-Hamilton Pleasure Scale (SHAPS) (n.s.).

### 3.3. Traditional Chinese Medicine Assessments

The results of the traditional Chinese medicine assessments of patients from this study have been published previously [69]. 

## 4. Discussion

In the current pilot study we examined the efficacy of acupuncture treatment for patients with schizophrenia living in the community. Outcome measures included three different domains—sleep measures, clinical and emotional measures, and traditional Chinese medicine assessments. The results following acupuncture treatment indicate a significant improvement in objective sleep measures collected by actigraph device, among them sleep onset latency, sleep percentage, mean activity level, wake time after sleep onset, mean number of wake episodes, mean wake episode, and longest wake episode. The treatment made patients fall asleep faster. They slept better, with less wake time during sleep and reduced activity level during sleep. As a result their sleep was more efficient. Moreover, the results of the current study revealed that, following acupuncture treatment, sleep percentage no longer met the criterion for insomnia (<85%) [70].

Interestingly, however, patients did not report any significant subjective improvement in their sleep. These findings raise the possibility of an optional discrepancy between subjective reported sleep measures and objective recorded measures. Many psychiatric disorders are evaluated both by objective and by subjective assessment tools to provide a general and multiangled perspective of the disorder examined. Yet, consistently arising discrepancies between different tools may suggest that these tools actually assess different phenomena. So the question is as follows: do subjective and objective sleep measures assess two distinct phenomena? In fact, several studies have shown that participant reports of their own sleep are only partly correlated to their objective sleep measures [46, 71–74]. The meaning of this difference between subjective sleep report and objective findings is far from being understood in terms of the biological factors involved, the clinical consequences, the treatment modality, and other factors as well. This subjective-objective discrepancy has been found repeatedly among different populations, such as posttrauma patients and elderly patients. Thus, findings revealing this discrepancy in patients with schizophrenia are in line with these studies. However, another explanation for this discrepancy of findings between objective and subjective assessments is the period of assessment of each measure. The objective measurements were taken over a brief period (one week at the end of the acupuncture treatment), while the patients may tend to subjectively assess their overall experience during the course of therapy, which they think may be ineffective. The subjective assessment results may also be heavily influenced by patients' own interpretation of their symptoms and experience as well as the wordings of the questionnaires.

Most importantly, these findings reinforce the influence of acupuncture on sleep measures, since the effect was found for the objective measures, implying that while the beneficial effect of acupuncture on sleep among patients with schizophrenia may exist, it may not be reportable. This makes the reliability of self-report with respect to sleep a questionable issue. These findings may be very important, since sleep difficulties are very common among patients with schizophrenia [19] and involve distress, malfunctioning, and impairment of quality of life, all directly related to sleep difficulties. Moreover, sleep difficulties and other domains of the psychopathology of schizophrenia, whether positive symptoms or symptoms such as anxiety and depression, have mutual and reciprocal relationships. Thus, sleep difficulties may play a crucial role in treatment resistance, partial remission, or relapse in schizophrenia.

Additionally, the results revealed an improvement in clinical and emotional assessments following acupuncture treatment. The core symptoms of schizophrenia, both positive and negative, as assessed by the Brief Psychiatric Rating Scale (BPRS) and by the Positive and Negative Syndrome Scale (PANSS), exhibited improvement compared to baseline following acupuncture treatments. Both the BPRS and the PANSS are commonly used scales among patients with schizophrenia for clinical diagnosis, for severity assessment, and for monitoring changes in the patient. Significant improvement on these scales shows a high correlation with clinical improvement, both objective and subjective.

This potential effect may be very important, especially in view of the unsatisfactory benefits of antipsychotic medications and the enormous burden of schizophrenia. This finding is concurrent with other studies that showed the beneficial effect of acupuncture on the core psychopathology of schizophrenia [36]. However, those studies had limited methodologies [36]. Likewise, the overall results of those studies are equivocal and insufficient [35]. 

Another clinical domain assessed was the domain of depression and anxiety. Depression and anxiety are very common in chronic schizophrenia and are connected with poor prognosis, greater distress, and higher rates of suicide [75–77]. It seems that acupuncture may not suffice as a sole treatment but rather can be used, as in this study, as an “add-on” for treating psychopathological domains other than the core domain of schizophrenia. Our study results revealed a significant beneficial effect of acupuncture on depression and anxiety levels, as assessed by the Calgary Depression Scale for schizophrenia (CDSS), by the State-Trait Anxiety Inventory (STAI), and by the Hamilton Anxiety Rating Scale (HAS). Because the symptoms of depression may overlap the negative symptoms of schizophrenia, we used the Calgary Depression Scale for schizophrenia (CDSS), which is used especially for assessing depression in the treatment of patients with schizophrenia. To summarize, the clinical outcome measures of the current study showed an overall improvement in depression, anxiety, and the core symptoms of schizophrenia. 

In this study we chose to use acupuncture as an “add-on” treatment, and all of the patients continued their medication treatment as usual. The reason for not using acupuncture as a sole treatment was the unsatisfactory efficacy of acupuncture alone for schizophrenia in studies published so far. Moreover, schizophrenia is a complex disorder with multiple areas of distress and dysfunction, among them intrapsychic, interpersonal, and occupational, with long-term residual symptoms and rehabilitation difficulties. On the other hand, conventional treatment for schizophrenia, even at its best when combining pharmacotherapy, psychotherapy, and rehabilitation, achieves unsatisfactory results and only partial remission. All this suggests that supplemental treatments are of the utmost importance. Acupuncture as a supplementary treatment seems to be a promising option. 

The study was carried out in the community with patients with schizophrenia living in hostels in “real life” situations with as few exclusion criteria as possible and without any limitations on the usual (pharmacotherapy, psychotherapy, and rehabilitation) treatment. There were no dropouts at any stage of the study. This zero dropout rate is unusual among general clinical studies and may signify high patient satisfaction with acupuncture treatment and low anxious or paranoid references, which would have been expected among patients with schizophrenia. 

Altogether, this pilot study may suggest a good setting for evaluating an “add-on” treatment, and the results may have implications for real treatment choices. This is in contrast to rigorously designed studies, in which conclusions concerning “real life and real patients" are hard to obtain, or at least problematic. 

This pilot study has several methodological limitations. Although the study used a within-subject design, it included a pre- and posttreatment evaluation but not a comparison with a control group, thus overlooking some other explanation for the effect. Therefore, the current study was vulnerable to placebo bias, either by participants or by therapists. Likewise, in this study, many statistical tests were performed, so there was a risk of committing type I errors. 

Remarkably, we found a consistent and interesting change in many qualities that were not systematically assessed, such as changes in patients' voices, general appearance, posturing, pace and movement, and vitality. Since all of these and many more qualities are familiar to TCM therapists, and since TCM treatment aims at relieving a broad spectrum of symptoms and distress, new assessment tools should be developed for similar future studies. Furthermore, since all these qualities seem to be connected to different aspects of well-being and quality of life, it may be equally important to develop new assessment tools for conventional pharmacotherapeutic studies.

In summary, to the best of our knowledge this is the first study to evaluate the efficacy of acupuncture on sleep in patients with schizophrenia using actigraph sleep measurement as an objective efficacy measure. Our results suggest that, for patients with schizophrenia, acupuncture treatment may have a possible beneficial effect on clinical core schizophrenic symptoms and on depressive and anxiety symptoms. Moreover, for patients with schizophrenia the results suggest a possible beneficial effect of acupuncture treatment on objective sleep but not on the patients' subjective experience in the short term while the patients are undergoing eight weeks of therapy. These findings need to be validated in future studies; that is, large randomized controlled trials are required to assess the efficacy of acupuncture treatment for insomnia among patients with schizophrenia.

The results of the current study demonstrated the potential short-term benefits of acupuncture. Future studies should examine whether these benefits will be maintained after cessation of therapy or whether acupuncture must be continued forever to maintain the benefits. Therefore, future studies are needed to validate the benefits of long-term acupuncture treatment. Since the results of the current study revealed that no patient experienced any adverse effects throughout the course of therapy, there is definitely room to check the feasibility of long-term acupuncture treatment among patients with schizophrenia.

## Figures and Tables

**Table 1:** Objective and subjective sleep measures (means and standard deviations) after each phase of the study and analysis of the comparisons between them.

Objective and subjective sleep measures	Baseline level Mean (SD)	Last week of acupuncture treatment Mean (SD)	*t*	*P* value
Total time in bed (minutes)	480.98 (±97.52)	475.39 (±96.82)	0.36	0.722
Sleep onset latency (minutes)	26.63 (±26.71)	14.32 (±12.81)	2.21	0.040
Sleep percentage (%)	84.20 (±10.76)	89.86 (±7.14)	2.90	0.010
Mean activity level	20.04 (±9.43)	13.26 (±7.63)	3.20	0.005
Wake time after sleep onset	67.43 (±45.86)	46.58 (±32.64)	2.92	0.009
Mean number of wake episodes	7.21 (±4.81)	5.42 (±2.31)	2.26	0.040
Mean wake episode (minutes)	3.76 (±1.62)	2.85 (±1.54)	2.49	0.023
Longest wake episode (minutes)	25.71 (±19.20)	18.16 (±11.55)	2.87	0.010
Subjective Sleep Quality (MSQ questionnaire)	3.64 (±1.15)	3.42 (±1.09)	2.152	0.187
Subjective Sleep Quality (PSQI questionnaire)	0.95 (±0.53)	0.80 (±0.54 )	1.632	0.119

**Table 2:** Emotional and clinical measures (means and standard deviations) after each phase of the study and analysis of the comparisons between them.

Emotional and clinical measures	Baseline level Mean (SD)	Last week of acupuncture treatment Mean (SD)	*t*	*P* value
Brief Psychiatric Rating Scale (BPRS)	2.49 (±0.81)	2.14 (±0.74)	6.67	0.001
Psychopathology score (PANSS)	2.96 (±0.98)	2.65 (±0.94)	8.26	0.001
Positive general symptoms (PANSS)	2.44 (±1.01)	2.04 (±0.88)	5.23	0.001
Negative general symptoms (PANSS)	3.62 (±1.20)	3.47 (±1.20)	2.97	0.008
General symptoms (PANSS)	2.83 (±0.95)	2.43 (±0.93)	7.96	0.001
Depression level (BDI questionnaire)	14.66 (±11.89)	12.01 (±11.61)	1.79	0.088
Depression level (CDSS questionnaire)	7.16 (±6.43)	4.21 (±4.90)	5.04	0.001
State Anxiety level (STAI questionnaire)	2.27 (±0.52)	2.12 (±0.48)	2.98	0.008
Anxiety level (HAS questionnaire)	1.21 (±0.68)	0.73 (±0.49)	5.96	0.001
Quality of Life level (QLESQ questionnaire)	3.60 (±0.58)	3.70 (±0.55)	−2.08	0.051
Anhedonia level (SHAPS questionnaire)	3.49 (±0.34)	3.41 (±0.45)	1.17	0.257
